# Draw‐Spinning of Kilometer‐Long and Highly Stretchable Polymer Submicrometer Fibers

**DOI:** 10.1002/advs.201600480

**Published:** 2017-05-12

**Authors:** Suiyang Liao, Xiaopeng Bai, Jianan Song, Qingyun Zhang, Jie Ren, Yusen Zhao, Hui Wu

**Affiliations:** ^1^ State Key Laboratory of New Ceramics and Fine Processing School of Materials Science and Engineering Tsinghua University Beijing 100084 China

**Keywords:** alignment, draw‐spinning, nylon, polymer fibers, stretchable fibers

## Abstract

**A new method is developed to directly spinning perfectly uniaxial fibers** in an ultrafast manner. Besides, this method can tune the fibers' diameter through adjusting processing parameters such as the feeding rate of precursors. Uniaxial nylon 66 fibers prepared via this method show superior mechanical properties due to the alignment in each level of the structure.

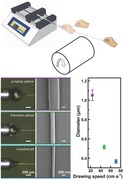

In this study, we report draw‐spinning method that can produce aligned polymer ultrathin fibers with diameter down to 200 nm. Apart from creating perfectly aligned fibers, this method can draw fibers at a high speed and can thus reorient molecular chains. Tensile tests show that nylon 66 fiber bundles fabricated by draw‐spinning exhibit >400% elongation at break and ≈250 MPa strength, which are superior to the corresponding values for conventionally fabricated fibers and bulks (usually <30% and <100 MPa, respectively). The superior performance can be attributed to the relative motions of building blocks in each of the four levels of the structure: the bundle, the fibers within the bundles, microfibrils within the fibers, and aligned molecular chains within the microfibrils. We confirm that each level within the hierarchy is highly aligned and propose a “tetra‐slip” system.

Fibers have been existing inside plants and playing structural roles to give mechanical support in harsh living conditions and produced by animals for specific applications.^1^, ^2^, ^3^ For example, to withstand huge bending forces from the wind, the inner fibrous structure within bamboos evolves to be highly uniaxial and various spider silks can perform a variety of roles such as webs and draglines.^4^, ^5^ Fibers of plants, such as flax, hemp, and jute, have been applied to daily usage.^6^ With the development of chemical synthesis, polymers such as nylon and kevlar were invented and fabricated into structural fibers. Except for direct applications, carbon fibers and glass fibers can be made into composites for reinforcement. Regardless of their component, fibers with superior mechanical performance have been a persistent topic. Apart from facilitating the evolution of component materials, adjusting the structure can be effective as well.^7^

In this regard, fiber diameter and alignment are two of the key factors. However, combining the structural advantages of these factors necessitates the improvement in fiber spinning methods.^8^ Industrial approaches, such as wet spinning, dry spinning, gel spinning, and melt spinning, are relatively fast, but the fiber products usually have diameters of dozens or hundreds of micrometers. Although direct drawing fibers from the precursor is quite simple, it has a low yield rate, and thus it has a low practical value.^9^, ^10^, ^11^, ^12^ Electrospinning, which originated from the 1940s, is probably the most well‐established processing method for nanofibers.^13^ Researchers have attempted to obtain aligned fibers by modifying the conventional setup of this process.^14^, ^15^, ^16^, ^17^, ^18^ However, due to the electrostatic repulsion among the charged fiber segments, the general products of electrospinning are nonwoven mats.^19^ Thus, the previous work rarely attained perfect alignment and precise positioning of the fibers. Except mechanical performance,^20^ profound practical values of aligned fibers lie in various fields. For example, in tissue engineering, highly aligned fibers can be applied in cell growth and drug delivery.^21^, ^22^ Periodic structures exhibit structural colors and generate fascinating applications in photonic crystals, and surfaces can be tuned to be hydrophobic by coating nanofibers.^23^ Generally speaking, aligned fibers can probably play a part in most anisotropic applications. Plenty work has been done to improve the mechanical performance of nylon 66 by adding other tougher materials. MWCNT (Multi‐wall Carbon Nanotube)‐strengthened uniaxial electrospun nylon 66 fibers exhibit elongation at break of 130% and ultimate strength of 100 MPa,^24^ which are six and nine times higher than those of the crosslink neat nylon 66 electrospun fibers.^25^ Other significant efforts include glass fiber‐reinforced nylon 66 via injecting molding and electrospinning nylon 66/organoclay nanocomposite.^26^, ^27^

In this communication, we present the general procedures of draw‐spinning and describe how it controls the diameter of aligned fibers; then, taking this structural advantage, we prepared nylon 66 and polyethylene oxide (PEO) fibers with different diameters for tensile tests; in the Experimental Section, we analyzed the alignment within each level of the tested bundles and proposed a “tetra‐slip” model to explain the mechanical results.

The basic setup for draw‐spinning is composed of two main parts. One part is a syringe that serves as the reservoir for the precursors and is loaded on a syringe pump, and the other is a collector, which is typically a rotating substrate (**Figure** **1**A; Figure S1, Supporting Information). The rotating substrate provides a force to draw fibers directly from the reservoir. To supply on demand, raw material feeding is controlled by the syringe pump to balance the take‐up speed. As for the collector, a 2D plate, a cylindrical roller, or even an object with any shape can be used to provide support for draw‐spun fibers. As shown in Figure 1B, a roller with a diameter of ≈16 cm was used in our experiment to collect the draw‐spun fibers. To initiate draw‐spinning, the precursor was prepared first. It was then loaded to a syringe, and the syringe with the precursor is placed in the syringe pump. While the pump is switched on, the liquid pool on the nozzle was manually drawn into a fiber. The rotating substrate was connected afterward. The draw‐spinning process stabilizes when the combination of the processing parameters are optimized. Continuously spinning PEO fibers at 1 m s^–1^ for 90 min resulted theoretically in one ≈5 km‐long submicrometer fiber (Figure 1C; Movies S1 and S2, Supporting Information). The fibers are highly aligned and the diameters are uniformly distributed. Two intrinsic properties of the precursor must be considered to reach an acceptable spinability. One is the molecular weight of the polymer, and the other is the surface tension of the solvent. High molecular weight ensures the entanglement of long molecular chains in less concentrated polymeric solutions. Fibers are formed only when the entangled molecular chains are consecutively drawn in this method, as poor entanglement caused either by low molecular weight or by low concentration reduces spinability. Another reason for the breakage of the fibers is surface tension. Solvents with low surface tensions or surfactants, such as sodium dodecyl sulfate and Triton X‐100, can be used to prevent breakage. The state of the precursor also influences the surface tension. Most organic solvents are volatile, but rapid solvent evaporation could unfavorably affect spinability. When the solvent evaporates, the solid residue could disrupt the feeding of the liquid precursor in the nozzle. In addition, the dried cone is extremely tough to be drawn into the fibers. Adjusting the feeding rate can balance the take‐up speed such that no redundant material accumulates on the nozzle. Moreover, the setup is highly adaptable. Using four‐nozzle needles can increase the production speed fourfold (≈5 m s^–1^ in Figure S2, Supporting Information).

**Figure 1:**
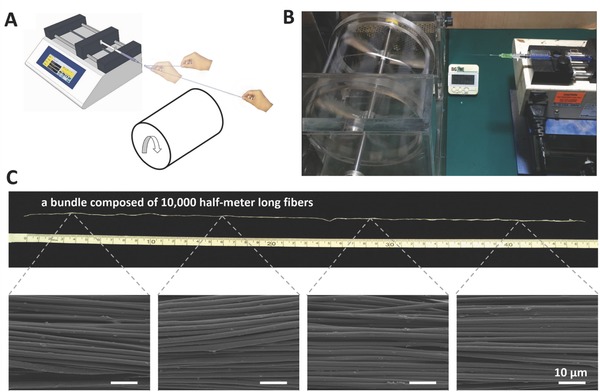
Basic setup, products, and procedures of draw‐spinning. A) Schematic illustration of the basic draw‐spin setup. B) Digital photo of the actual draw‐spin setup in our lab. C) Fiber bundle removed from the roller (The lower row: SEM images of the as‐prepared fibers).

Spinning parameters influence the morphology of the fiber in most cases. The influential parameters include the voltage in electrospinning to control the drawing force, the molecular weight, the concentration of the precursors, the travelling distance, and the flux rate.^28^, ^29^, ^30^, ^31^, ^32^ In the present study, we evaluated two draw‐spinning parameters that affect the size of the fibers, the drawing speed and the precursor concentration.

The draw‐spinning process is a balanced result between feeding and consuming rates of the precursors. Under the same feeding rate, the consuming rate can fluctuate within a certain range while maintaining spinability. Deformation of liquid pools and change in fiber diameter can be observed when the feeding and consuming rates are mismatched. Investigations on the diameter's dependence on drawing speed were performed on PEO fibers. The substrate was a silicon wafer attached to a roller with a diameter of ≈3 cm. While maintaining the feeding rate at 0.13 mL h^–1^ and changing the rotating speed, we obtained the magnified digital photos of the deformed liquid cones. As shown in **Figure** **2**A, from top to bottom, the rotating speeds are 150, 300, and 450 RPM (the take‐up speeds were 23.6, 47.1, 70.7 cm s^–1^, respectively). As a result, the shape of the liquid pool on the 0.06‐mm nozzle transformed from “jumping sphere” to “constant jet.” When the amount of the precursor is fixed, an increased rotating speed directly leads to an increase in production rate, resulting in thinner fibers, as shown in the scanning electron microscopy (SEM) images in the right‐hand column, and the scatter plot regarding the statistics are depicted in Figure 2B and Figure S3 (Supporting Information). Movie S3 (Supporting Information) provides a dynamic demonstration of the balance and mismatch during draw‐spinning. During traveling from the nozzle to the substrate or after depositing on the collector, the solvent in the as‐prepared fibers tends to evaporate, and the solute thus becomes the final product. The diameter of the draw‐spun fibers was hypothesized to be closely related to precursor concentration. To test this hypothesis, highly concentrated (3.2 wt%) PEO/acetonitrile precursor was prepared. By consecutively performing the “spin‐dilute” operation several times, the lowest spinable concentration was reached at ≈0.5 wt%, and several data points were acquired, as shown in Figure 2C. A positive correlation between the diameters of the draw‐spun fibers and the polymeric ratios of the precursor was observed, and the diameter can be adjusted from several micrometers to ≈200 nm. Profound meanings in patterning arrays and grids are present. Massive efforts have been made to overcome the randomness of electrospinning. The lowest pitch between the near‐field electrospun fibers is ≈5 µm, but at 2 mm s^–1^.^33^ A high speed version of this method can reach 0.5 m s^–1^, but the lowest spacing is 100 µm.^34^ Draw‐spinning can achieve each of this result without compromising the other. To arrange fibers into an array, we used another syringe pump to introduce translational motion into the system. By mounting the motor‐powdered substrate onto the syringe pump, we integrate rotating motion and linear motion to wind one fiber into an array (Figure S4 and Movie S4, Supporting Information). SEM images of the perfectly aligned PEO fibers with different spacings (6, 12, and 18 µm) are shown in Figure 2D. Another proof for the recurring structures is the structural colors caused by the diffraction grating effect.^35^ This result also shows the potential of the method in coating (Figures S5 and S6, Supporting Information). After an array is prepared, rotating the substrate and spinning again deposit another layer of fiber array on the previous array, resulting in a grid (Figures S7 and S8, Supporting Information). Besides polymer fibers, we also achieved the patterning of brittle ceramic fibers and successfully assembled metal fibers into grids (Figure S9, Supporting Information). One of the envisioned field of fibers, especially the fiber mesh is flexible electronics.^36^, ^37^, ^38^ The potential advantage is to reach bulk performance in conductivity, especially when the junctions are welded together and while the smallest amount of raw materials are used, improving the transparency of the electrode. In addition, because mechanical strength is strongly related with structures, the square holes are expected to deform in order to survive external stress. Thus, the mesh must be a good starting point toward a flexible, stretchable, and transparent electrode.^39^, ^40^, ^41^

**Figure 2:**
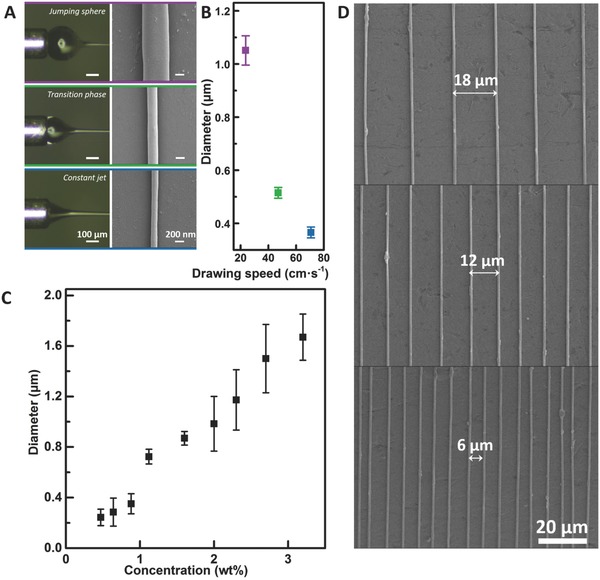
Diameter control and fiber arrays. A) Diameter and feeding rate. Magnified digital photos of the deformed cones caused by the “feeding‐consuming” mismatch (left column) and SEM images of the corresponding products (right column). B) Scattered points showing the diameter of the fiber products fabricated under corresponding processing parameters. C) Upward trend of the diameter change when polymeric concentration increases. D) Fiber arrays of varying spacings: 18, 12, and 6 µm.

As mentioned previously, spinability can be maintained even when a mismatch between feeding and consuming is present, although it influences the diameter of the fibers. Thus, we fixed the flux rate while changing the rotating speed to produce a bundle. With the flux rate and fabricating time fixed, we produced two bundles consisting of fibers with different diameters. Specifically, we used a roller to collect nylon 66 fibers (mixed with PEO at the weight ratio of 10:1). The product is a fiber “ring,” as removed from the roller. It was then folded twice and twisted. Draw‐spun nylon 66 fiber bundle can stretch from its original length of 5–25 cm (**Figure** **3**A; Movie S5, Supporting Information). To compensate the fourfold increase along the axis, the average diameter shrank from 3.0 to 1.6 µm (Figure 3B). The notable mechanical properties are the Young's modulus, the tensile strength and the elongation at break. By contrast, the elongations at break of the electrospun nylon 66 fibers are mostly below 100%. Since the chemical components of the samples in the published work are different from our samples, we prepared nylon 66 thick fibers (diameter: ≈75 µm) following the wet‐spinning procedures for tensile tests. As shown in Figure 3C, the elongation and the strength of our samples can reach 400% and 230 MPa, respectively, superior to most published results, plotted as scattered points. The diameter mainly has influences on the strength and the modulus. The faster spun samples exhibit the strength three times higher than its slower spun counterparts and the modulus can be improved from 0.81 to 1.35 GPa. In Figure 3D, comparable results can be found in draw‐spun PEO fibers. We also investigated the mechanical performances of fibers with different proportion of nylon 66 and PEO, and found that the strength increases with the addition of nylon 66 (Figure S10, Supporting Information). Tolerance against crack propagation is one of the mechanical advantages of the fiber bundles. When the bulk counterparts are stretched, the stress concentration starts when the components are interlocked, and then cracks propagate to cause failure. However, failure of a single fiber in a bundle does not lead to the breakdown of the entire system. The support for this theory can be found from the “staircases” in the strain–stress curves shown in the left plot of Figure 3D.

**Figure 3:**
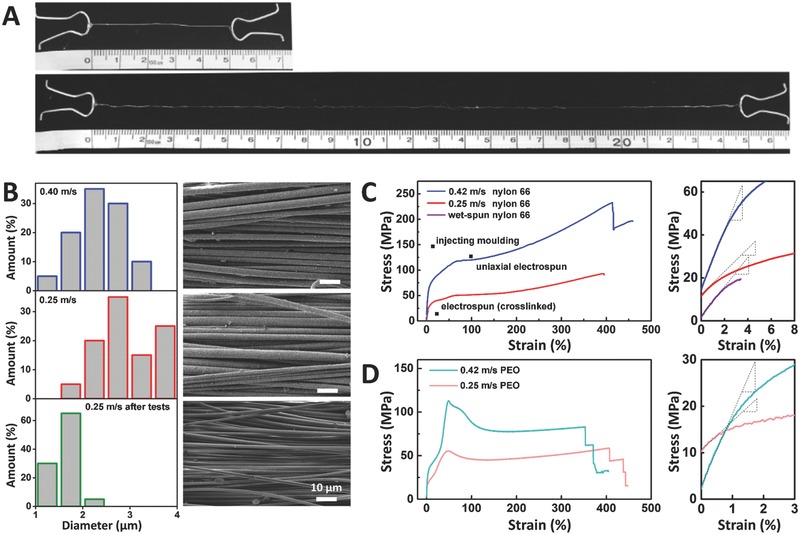
Tensile tests of draw‐spun nylon 66 fibers. A) Photos of a bundle before and after the test. B) Diameter distribution of fibers: tunable average diameter and the shrinkage of diameter after tensile tests. C) Strain–stress curves of nylon 66 fibers. Right: Young's modules region. D) Strain–stress curves of PEO fibers. Right: Young's modules region.

The reason for the huge discrepancy between the actual and theoretical mechanical performance is the folding of the polymeric chains. As soon as they are extruded, industrial spun fibers immediately go through a drawing process to increase their strength but at a sacrifice of stretchability. For a better analysis of the mechanical advantage, we revealed the four‐level alignment and propose a “tetra‐slip” system. On macroscopic levels, slipping level‐1 occurs between the four strands, and inside each strand, slipping level‐2 occurs between the draw‐spun fibers (**Figure** **4**A,B). At microscopic levels, the nylon 66 microfibrils are oriented, most likely because of the shear force when they are drawn. By selectively dissolving PEO in the mixture using acetonitrile, we showed the shape of the nylon 66 microfibrils. Figure 4C shows that in draw‐spun products, the microfibrils are elongated along the shear drawing force. For comparison, we cast the same precursor on glass films by dipping, and after the selective dissolution, the microfibrils are randomly distributed. Fourier transformed infrared spectroscopy was conducted to verify the effectiveness of the selective dissolution (Figure S1, Supporting Information). During stretching, slipping occurs between the oriented microfibrils. In conventional materials, microfibrils interlock each other. Finally, innermost slipping is present between the aligned linear molecular chains. Raman spectroscopy characterized the orientation of the molecular chains.^42^ The Roman intensity was acquired twice for each sample, which was rotated 90 °C horizontally for the second measurement. In this way, the fiber axis was positioned parallel or perpendicular to the exciting laser beam. A large difference between the two sets of data indicates high heterogeneity or intensive orientation of the molecular chains. Figure 4D shows the alignment of the molecular chains in the following order: cast films, as‐prepared draw‐spun fibers, and draw‐spun fibers after tensile tests. During stretching, the components in the tetra‐slip system relocate and reorient, thus effectively avoiding stress concentration.

**Figure 4:**
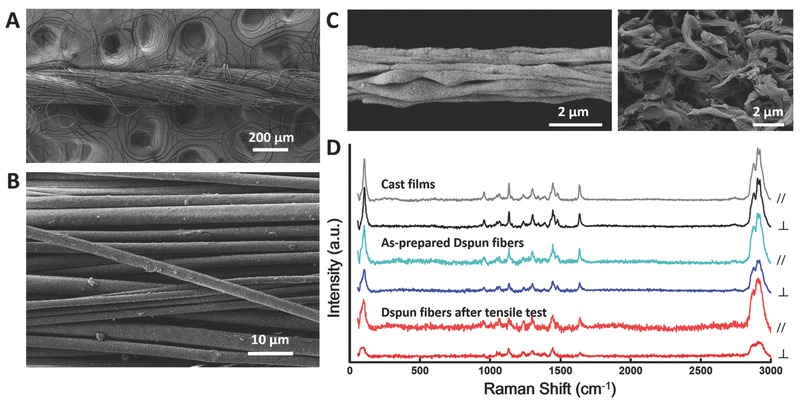
Tetra‐slip system as an explanation for the mechanical advantages of draw‐spun fiber bundles. A) Twisted four strands. B) Perfectly aligned fibers. C) Selective dissolution of PEO for nylon 66 microfibrils. Left: oriented nylon 66 microfibrils in draw‐spun fibers; Right: randomly distributed microfibrils in cast films. D) Raman proof of molecular orientation.

To conclude, draw‐spinning possesses several advantages over current processing methods for micro‐/nanofibers. First, single continuous fibers with limitless length and tunable diameter can be fabricated through this method. Second, the fibers can be arranged into arrays and meshes. Third, this method is adaptable and versatile, and it can be integrated with current processing methods or followed with well‐established post‐treatments seamlessly to create novel structures and expand the draw‐spinable systems. Small fiber diameter and better fiber alignment improve mechanical properties. We conducted tensile tests on draw‐spun nylon 66 fiber bundles to investigate the influence of fiber diameter on mechanics. Because of the structural advantages, our product is tenfold higher than most nylon 66 samples when elongated, demonstrates superior performance in strength. The mechanical improvements can be explained by our tetra‐slip system.

## Experimental Section

*Sample Preparation*: Polymer solution containing a certain weight ratio (ranging from 4.0 to 0.8 wt%) of PEO (*M*_v_ = 8 000 000, Sigma‐Aldrich) was prepared using acetonitrile (Analytical Reagent, Sinopharm Chemical Reagent Co., Ltd, China) as the solvent. The nylon 66 precursor was prepared using nylon 66 and PEO (three weight ratios were chosen in our experiment, 10:1, 6:1, and 3:1) as the solutes. Nylon 66 was added into the ≈1 wt% PEO/formic acid (chemically pure, Sinopharm Chemical Reagent Co., Ltd, China) and stirred until dissolution.

*Draw‐Spinning Procedures*: Precursors were loaded into a syringe, which was then mounted onto a syringe pump. A rotating roller was used as the collector of draw‐spun fibers and by manually drawing the liquid cone on the nozzle into a fiber onto the rotating roller, the precursor can be constantly spun into fibers. To construct arrays, the translational motion was added to the rotating roller.

*Sample Characterization*: Morphology and microstructures of fibers were observed using a field‐emission electron scanning microscopy (LEO‐1530, Zeiss, Germany). Energy Dispersive Spectroscopy signals of the fiber arrays were collected using detectors from Oxford Instrument (X‐Max^N^ Silicon Drift Detector). Optical transmittance was measured using a UV–vis spectroscopy (SHIMADZU UV‐2600). Fourier‐transformed infrared spectrum was obtained using an IR spectrometer from Bruker Corp (VERTEX 70v). Powder samples were mixed with KBr at weight ratio of 1:100; bulk samples were examined with the help of ZnSe as the attenuated total reflectance crystal. Raman intensity was performed using a Raman spectrometer with a 600 gr mm^–1^ grating and the laser emitting at 532 nm (LabRAM HR Evolution, HORIBA Jobin Yvon, French).

*Tensile Tests*: Tensile tests were performed using a Zwick universal testing machine, Zwick Roell Z005, under room temperature and at moisture of ≈30%. The effective area of the samples was calculated via multiplying the average cross sectional area of an individual fiber by the overall amount of fibers. For each sample, more than three measurements were conducted to ensure the repeatability and the plotted curves are representative. The test speeds were 130% strain per minute. Results at other speeds are available in Supporting Information.

## Supporting information

Supplementary

Supplementary

Supplementary

Supplementary

Supplementary

Supplementary
