# Supplementary material for: Draw‐Spinning of Kilometer‐Long and Highly Stretchable Polymer Submicrometer Fibers
Source: Adv Sci (Weinh). 2017 May 12;4(9):1600480. doi: 10.1002/advs.201600480 (PMC5604378; doi:10.1002/advs.201600480)
Supplement: Supplementary file 1 — Supplementary [file ADVS-4-na-s001.pdf]

Copyright WILEY-VCH Verlag GmbH & Co. KGaA, 69469 Weinheim, Germany, 2017.

ADVANCED SCIENCE Open Access

Supporting Information

for *Adv. Sci.,* DOI: 10.1002/advs.201600480

Draw-Spinning of Kilometer-Long and Highly Stretchable Polymer Submicrometer Fibers

*Suiyang Liao, Xiaopeng Bai, Jianan Song, Qingyun Zhang, Jie Ren, Yusen Zhao, and Hui Wu**

Copyright WILEY-VCH Verlag GmbH & Co. KGaA, 69469 Weinheim, Germany, 2016.

Supporting Information

Draw-spinning of kilometer-long and highly stretchable polymer submicron fibers

*S. Liao, X. Bai, J. Song, Q. Zhang, J. Ren, Y. Zhao, and H. Wu**

In this file:

Supplementary Figures 1~11

Supplementary Movies (1~5): Captions and Descriptions

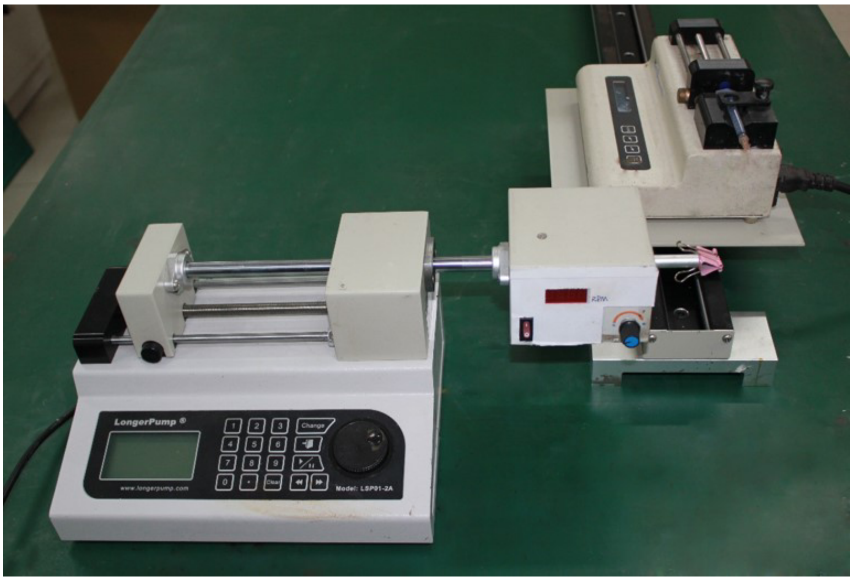

Figure S1. Adapted D-spin setup for fabricating arrays. By mount the motor-powered rotatable substrate onto another syringe pump, translational motion can be introduced into the system to assemble fibers into arrays.

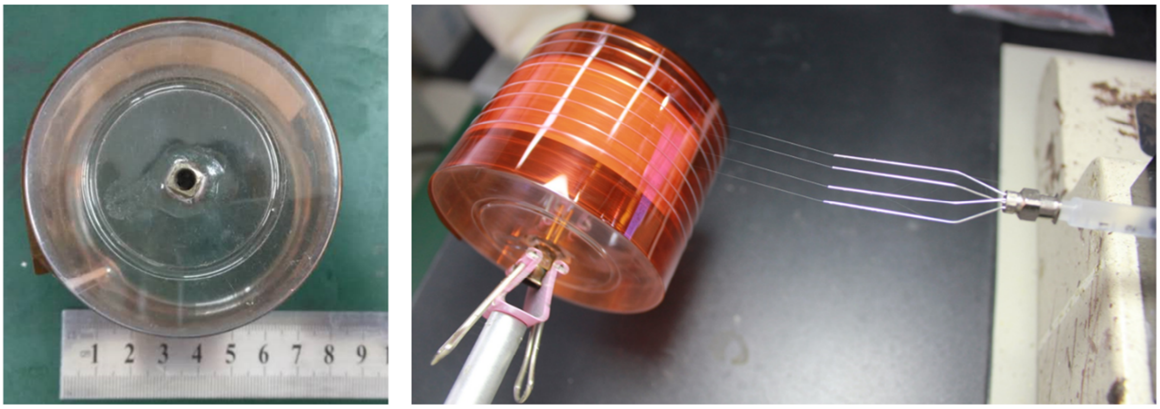

Figure S2. Using the multi-nozzle syringe to increase the production rate. Rotating the roller (diameter: 8 cm) at 300 RPM and drawing fiber from a four-nozzle needle, the overall production rate is ~5 m/s.

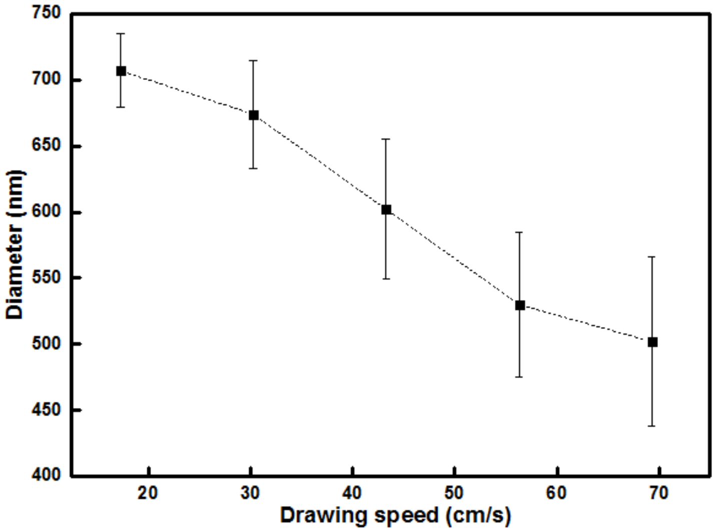

Figure S3. The downward trend of fibers' diameter when the drawing speed increases.

(concentration of the PEO/acetonitrile system: 0.6 wt%, feeding rate: 0.10 mL/h)

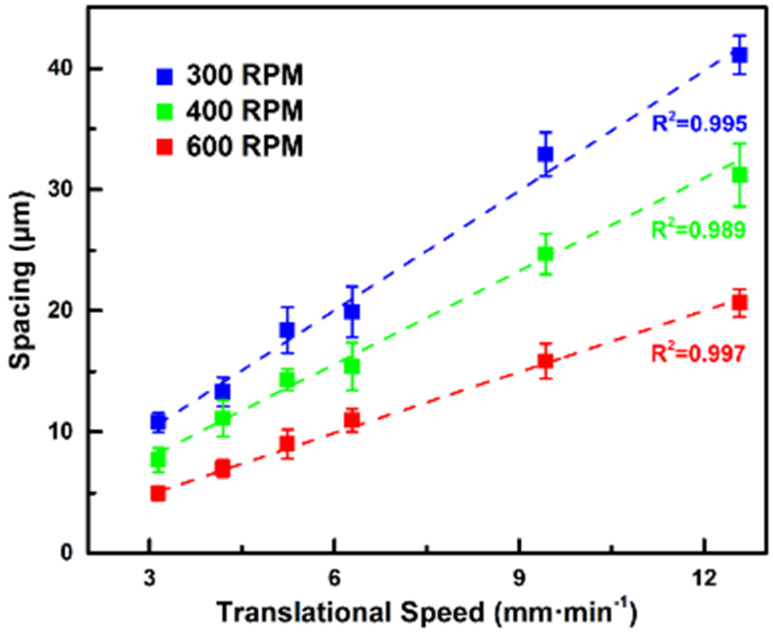

Figure S4. Scattering plot and linear fitting of spacings' dependence on rotational speed and translational speed. Three different rotating speeds, 300, 400 and 600 RPM were chosen to confirm the spacing control ability. Six data points were calculated from corresponding SEM images for each rotating speed and depicted with error bars. Apparently, though with systematic errors, the spacings' dependence on the flux rate follows a linear rule.

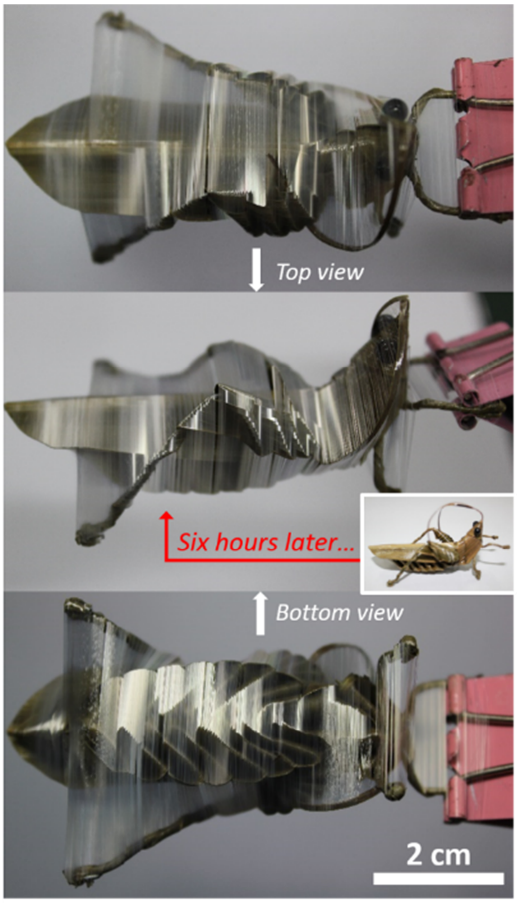

Figure S5. Using a "cricket" to collect the Dspun PEO fibers for about six hours to be totally enwrapped. Extra attention should be paid to the variable shape of the "cricket", which greatly challenges the stability of the method.

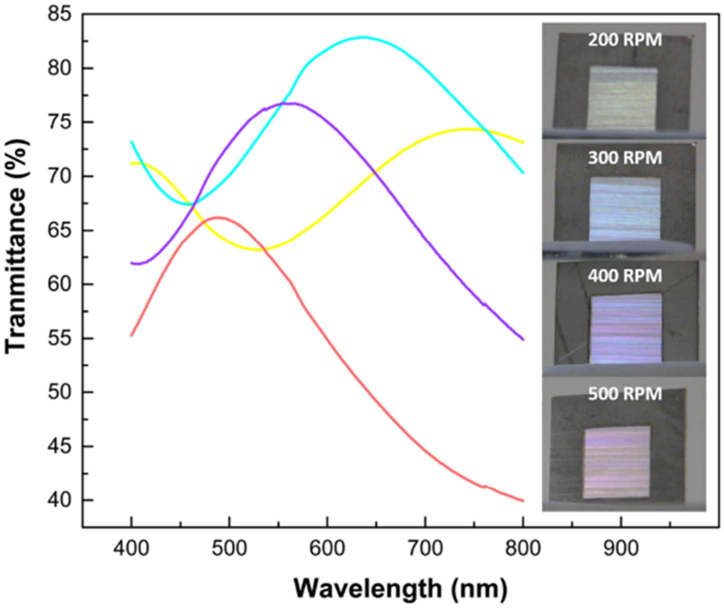

Figure S6. Iridescence/structural colors of periodic Draw-spun fiber arrays.

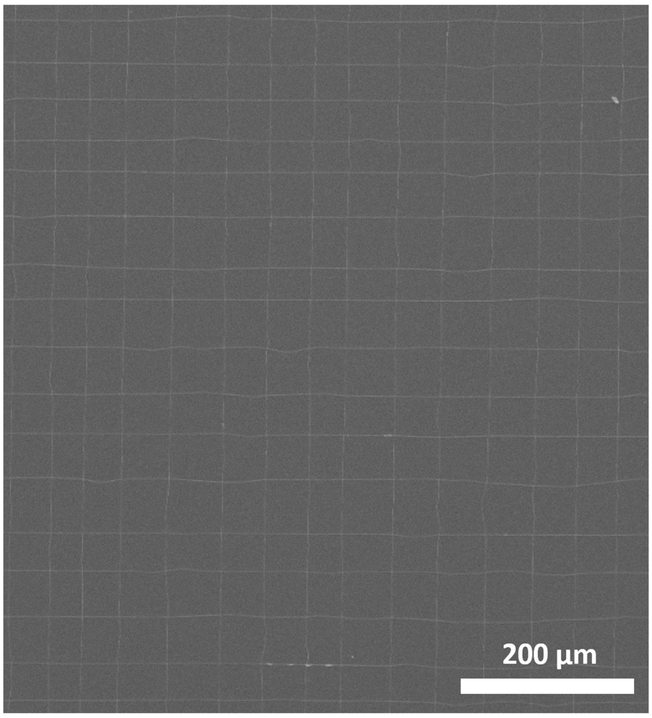

Figure S7. A 16 × 16 mesh based on PEO fibers. The actual product is a 1 cm ×1 cm Si wafer.

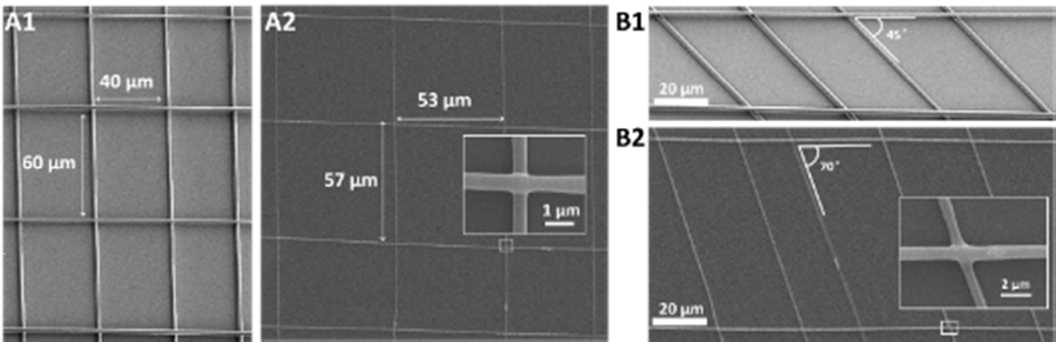

Figure S8. Fiber meshes with tunable shapes and junction states. (A1 and A2) a rectangular mesh with ovelapped juncitons based on PVP fibers (left) and a square mesh with fused junctions based on PEO fibers. (right) The inset is the zoomed-in picture of the junctions. (B1 and B2) PVP fibers intersected at the angle of 45 °, with overlapped junctions. (top) PEO fibers intersected at the angle of 70°, with fused junctions. (bottom)

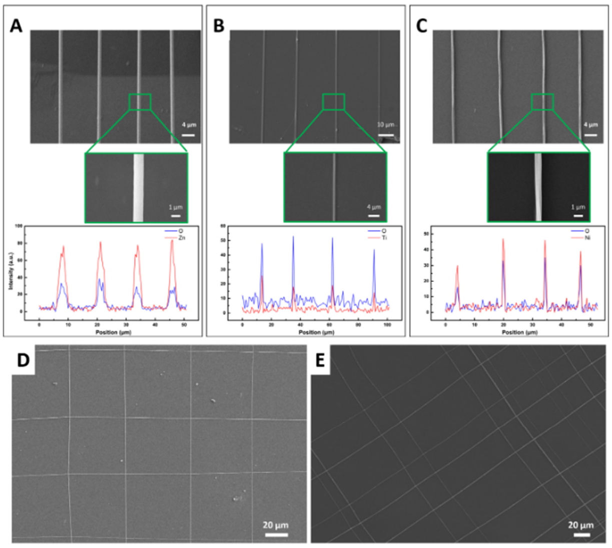

Figure S9. Ceramic and metal fibers realized by draw-spinning. Other than just polymer fibers, ZnO, TiO_2 and NiO fibers were also prepared and assembled into arrays, with EDS line scannings proving both the element constitution and precise positioning. (A, B and C); Meshes: TiO_2 and Ag (D and E)

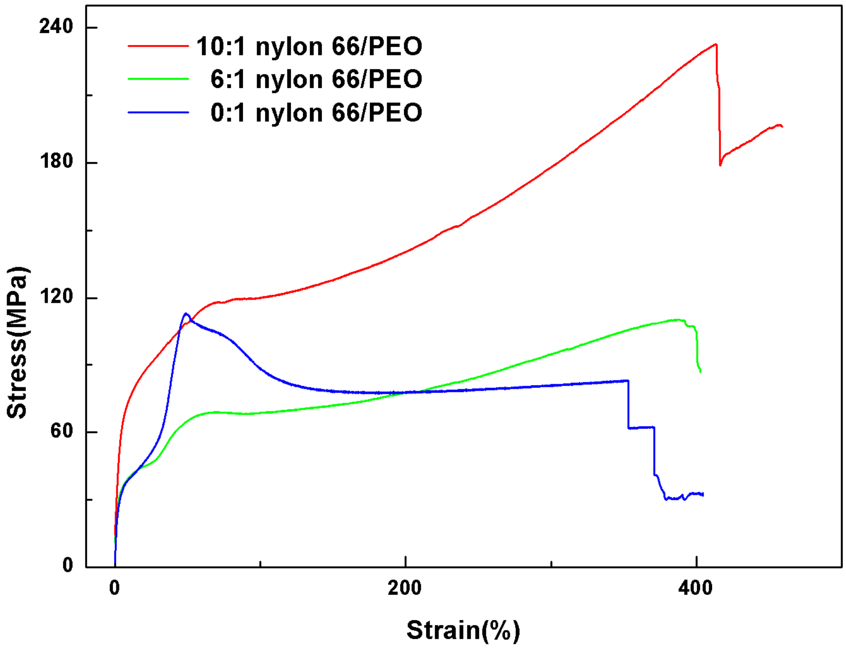

Figure S10. The results of tensile tests on fibers of different nylon 66/PEO ratio.

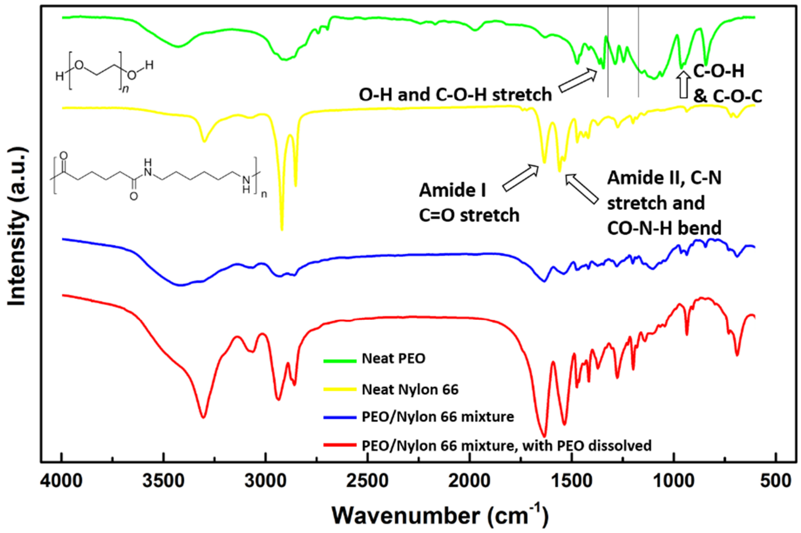

Figure S11. FTIR proof of selectively dissolving PEO in nylon 66. Using the system of PEO/nylon 66 precursor and selectively dissolving PEO by soaking the products in acetonitrile, the deformation of the remained nylon 66 microfibrils proved the existence of the shear force during the high-speed draw-spinning process.

Movie 1. Continuous production at 1 m/s for 90 min without breakage. This video demonstrates the very basics of draw-spinning. A roller (diameter: 16 cm) rotates at ~130 RPM to collect fibers for 90 min without breakage. Theoretically, the final product is one microfiber with the length of >5 km.

Movie 2. Ultrafast draw-spinning at 2.5 m/s

Movie 3. The balance and mismatch of feeding and consuming. By keeping the major processing parameters fixed and changing the rotating speed from 150 RPM to 600 RPM, we found that the shape of the liquid cone changes from “jumping sphere” into “constant jet”, resulting from the “feeding-consuming” mismatch.

Movie 4. Assembling fibers into arrays. By mounting the motor/substrate onto another syringe pump, we introduce translational motion into the system. As a synergetic result, fibers can be arranged into arrays with definable spacings.

Movie 5. Tensile test. This speed-up video demonstrates the tensile test at 130 $\%\cdot min^{-1}$.
